# Aquaporin 1 Is Involved in Acid Secretion by Ionocytes of Zebrafish Embryos through Facilitating CO_2_ Transport

**DOI:** 10.1371/journal.pone.0136440

**Published:** 2015-08-19

**Authors:** Jiun-Lin Horng, Pei-Lin Chao, Po-Yen Chen, Tin-Han Shih, Li-Yih Lin

**Affiliations:** 1 Department of Anatomy and Cell Biology, School of Medicine, College of Medicine, Taipei Medical University, Taipei, Taiwan; 2 Department of Life Science, National Taiwan Normal University, Taipei, Taiwan; Institut National de la Recherche Agronomique (INRA), FRANCE

## Abstract

Mammalian aquaporin 1 (AQP1) is well known to function as a membrane channel for H_2_O and CO_2_ transport. Zebrafish AQP1a.1 (the homologue of mammalian AQP1) was recently identified in ionocytes of embryos; however its role in ionocytes is still unclear. In this study, we hypothesized that zebrafish AQP1a.1 is involved in the acid secretion by ionocytes through facilitating H_2_O and CO_2_ diffusion. A real-time PCR showed that mRNA levels of AQP1a.1 in embryos were induced by exposure to 1% CO_2_ hypercapnia for 3 days. In situ hybridization and immunohistochemistry showed that the AQP1a.1 transcript was highly expressed by acid-secreting ionocytes, i.e., H^+^-ATPase-rich (HR) cells. A scanning ion-selective electrode technique (SIET) was applied to analyze CO_2_-induced H^+^ secretion by individual ionocytes in embryos. H^+^ secretion by HR cells remarkably increased after a transient loading of CO_2_ (1% for 10 min). AQP1a.1 knockdown with morpholino oligonucleotides decreased the H^+^ secretion of HR cells by about half and limited the CO_2_ stimulated increase. In addition, exposure to an AQP inhibitor (PCMB) for 10 min also suppressed CO_2_-induced H^+^ secretion. Results from this study support our hypothesis and provide in vivo evidence of the physiological role of AQP1 in CO_2_ transport.

## Introduction

Aquaporins (AQPs) are expressed in a variety of water-transporting epithelia, such as the kidney, stomach, and small intestine and play an important role in facilitating water transport across cell membranes [1]. In addition to water, AQPs also transport small molecules such as urea and glycerol [1, 2]. Moreover, AQPs were shown to be permeable to gas molecules. For example, AQP1, AQP4, and AQP5 were found to have high permeability to CO_2_ [2, 3, 4]; AQP3, AQP8, and AQP9 were found to transport NH_3_ [5], and AQP1 and AQP4 were suggested to transport NO [6, 7].

AQP1 was the first channel protein identified to be a CO_2_ gas channel. It is highly expressed in tissues necessary for CO_2_ transport such as pulmonary capillaries, vascular smooth muscle, and red blood cells, supporting the hypothesis that AQP1 can function as a CO_2_ channel [2]. AQP1 expressed by oocytes or reconstituted into proteoliposomes was found to increase CO_2_ permeability [8, 9]. By measuring the exchange of ^18^O between CO_2_ and HCO_3_^−^, Endeward and colleagues [10] showed that CO_2_ permeability was reduced by 60% in AQP1-null human erythrocytes compared to wild-type erythrocytes. In addition, CO_2_ permeability was reduced in proximal tubules isolated from AQP1^−/−^ mice and loss of AQP1 also led to less HCO_3_^−^ reabsorption [11]. Those studies revealed the function of AQP1 in CO_2_ transport.

Zebrafish embryos have become an animal model for investigating ionocytes and transepithelial ion transport [12, 13, 14]. At least 4 subtypes of ionocytes were identified in the skin and gills of zebrafish, i.e., H^+^-ATPase-rich (HR) cells, N^+^/K^+^-ATPase-rich (NaR) cells, Na^+^-Cl^−^ cotransporter-expressing (NCC) cells, and K^+^ secretion (KS) cells. These ionocytes are responsible for several important functions including taking up Na^+^, Cl^−^, and Ca^2+^ from water to maintain body fluid homeostasis and secreting metabolic H^+^ and NH_4_^+^ into the surrounding water [12, 13, 14]. In zebrafish embryos, mRNA expression of AQP1a.1 by skin ionocytes was recently identified [15]. Functional studies with *Xenopus* oocytes demonstrated that zAQP 1a.1 can facilitate CO_2_ and NH_3_ diffusion across cell membranes [15]. Following that study, an eel AQP1a antibody was used to localize zAQP1a.1 in the basolateral membranes of zebrafish HR and NaR cells [16].

HR cells are responsible for Na^+^ uptake and acid secretion (H^+^/NH_4_^+^) through coordinating several transporters and enzymes, including apical H^+^-ATPase (HA), the Na^+^/H^+^ exchanger (NHE3b), *Rhesus* glycoproteins (Rhcg1), cytosolic carbonic anhydrase (CA2), and the basolateral anion exchanger (AE1b) and Na^+^/K^+^-ATPase (NKA) [14]. Loss-of-function experiments demonstrated the critical role of HA in secreting acid from apical membranes of HR cells [17]. Upregulation of HA expression and the accompanying acid secretion by HR cells were found in embryos subjected to metabolic acidosis [18]. The ammonia transporter, Rhcg1, was found to facilitate intracellular NH_3_ passage through apical membranes of HR cells [19, 20]. Na^+^ uptake was achieved through a coupling function of NHE3 and Rhcg1 [21]. CA2 was suggested to promote the reaction of CO_2_ hydration and generation of H^+^ and HCO_3_^−^ in HR cells [22]. The generated H^+^ and HCO_3_^−^ were respectively secreted into the external and internal environments via the apical HA/NHE3 and basolateral AE1b [23]. Based on the function and transport mechanism, HR cells were proposed to be analogous to mammalian A-type intercalated cells or proximal tubular cells [13, 14].

Because of localization of AQP1a.1 in basolateral membranes of HR cells and an in vitro experiment which showed that AQP1a.1 can enhance H_2_O/CO_2_ permeability of oocytes, we hypothesized that AQP1a.1 in HR cells is involved in acid secretion by facilitating H_2_O/CO_2_ diffusion from the circulation into HR cells. To test this hypothesis, we analyzed acute hypercapnia (1% CO_2_)-induced H^+^ secretion by HR cells with a scanning ion-selective electrode technique (SIET) and evaluated the influences of AQP1a.1-knockdown and an AQP inhibitor on H^+^ secretion. In addition, in situ hybridization was used to confirm the expression of AQP1a.1 by HR cells. A real-time polymerase chain reaction (PCR) was used to examine the influence of hypercapnia acclimation on the expression of AQPs in embryos.

## Materials and Methods

### Zebrafish and hypercapnic exposure

Adult zebrafish (AB strain) were reared in circulating tap water at 28°C with a photoperiod of 14 h of light/10 h of dark. Fertilized eggs were incubated in artificial normal water (NW). The NW contained (in mM) 0.5 NaCl, 0.2 CaSO_4_, 0.2 MgSO_4_, 0.16 KH_2_PO_4_, and 0.16 K_2_HPO_4_ (pH 7). All of the incubating solutions were prepared by adding various salts (Sigma-Aldrich, St. Louis, MO, USA) to double-distilled water. During the experiments, embryos were not fed, and the NW was changed daily to ensure optimal water quality. For hypercapnic experiments, zebrafish embryos were exposed to NW bubbled with 1% CO_2_ in an air mixture for 3 days to determine mRNA expressions of genes. The hypercapnic water was approximately pH 6.3. The experimental protocols were approved (no. 100027) by the National Taiwan Normal University Animal Care and Utilization Committee.

### Reverse transcription (RT)

Zebrafish embryos were collected and homogenized in Trizol reagent (Ambion, Woodward, TX, USA). Total RNA was purified following the manufacturer’s protocol. The total amount of RNA was determined at absorbances of 260 and 280 nm by spectrophotometry (ND-1000, NanoDrop Technology, Wilmington, DE, USA). All RNA pellets were stored at -20°C. For complementary (c)DNA synthesis, 5 μg of total RNA was reverse-transcribed in a final volume of 20 μl containing 0.5 mM dNTPs, 2.5 μM oligo(dT)_20_, 5 mM dithiothreitol, 40 units of an RNase inhibitor, and 200 units of SuperScript III RT (Invitrogen, Carlsbad, CA, USA) for 1.5 h at 55°C, followed by incubation for 15 min at 70°C. Thereafter, remnant RNA was removed by incubation with 20 units of *Escherichia coli* RNase H (Invitrogen) for 20 min at 37°C. For PCR amplification, 1 μl of cDNA was used as a template in a 25-μl final reaction volume containing 0.25 μM dNTP, 1.25 units of Gen-Tag polymerase (Genemark, Taipei, Taiwan), and 0.2 μM of each primer.

### Quantitative (q)RT-PCR

Expression levels of *aqp1a*.*1*(*zaqp1a*) (ENSDARG00000023713), *aqp3a* (ENSDARG00000003808.4), *aqp3b* (ENSDARG00000069518.4), *aqp4* (ENSDARG00000010565.7), *aqp7* (ENSDARG00000026787.8), *aqp8a*.*1* (*aqp8aa*) (ENSDARG00000045141.5), *aqp9a* (NM_001033096), *aqp10a* (ENSDARG00000007086.9), and *aqp12* (ENSDARG00000043279.6) mRNAs were measured by a qRT-PCR with a Roche Lightcycler 480 (Roche, Penzberg, Germany). The final reaction volume in each well was 10 μl, which consisted of 5 μl of 2x SYBR green master mix (Roche), 3.2 ng of cDNA, and 50 nM of primers. Standard curves for each gene were generated in the linear range, and the gene encoding ribosomal protein L13a (rpl13a; ENSDARG00000044093.4) was used as an internal control. Primer sets used for the qRT-PCR are shown in Table 1. The specificity of the primer sets was confirmed by the presence of a single peak in the dissociation curve analysis and by detection of a single band of the correct size by gel electrophoresis.

**Table 1:** Primers used for qPCR analysis.

Gene name	Primer sequence
***aqp1a*.*1***	
Forward	5’TCTATGACTTTCTGCTTTACCCA3’
Reverse	5’CGTTGACCTCATAATCAGTGGC3’
***aqp3a***	
Forward	5’TGGACCCCTACAACAACCCG3’
Reverse	5’TGCCATCCCACCATCAGC3’
***aqp3b***	
Forward	5’ATCCTGGAAAAGATGGCTCG3’
Reverse	5’CATTCTGCCAGTGCTTGTCTC3’
***aqp4***	
Forward	5’TGTCCTGACCCTGACCTGA3’
Reverse	5’GCTCCTTCTTCTCCAAATCC3’
***aqp7***	
Forward	5’AGCGGTTACGCCATCAATC3’
Reverse	5’GCCCCTAAAACTCCTCCAAT3’
***aqp8a*.*1***	
Forward	5’GAACGGAAGAACCAAAAGTCA3’
Reverse	5’GGTAAGAGGTCCAACCCAATAA3’
***aqp9a***	
Forward	5’TCATCACTCCAATCACAACGA3’
Reverse	5’CAGGCTGCTGTCCTCTTCTT3’
**a*qp10a***	
Forward	5’TTTAGGGGCTTACCTTGCTT3’
Reverse	5’CCAAATACGGTCAGAACTCCA3’
***aqp12***	
Forward	5’ATCGCTTACACCGCAAACA3’
Reverse	5’CGAGCCAGTAGACCAGTGAATA3’

### Whole-mount in situ hybridization

For in situ hybridization of aqp1a.1, primers (forward: 5’-AACACCAACACTCAAAACCCAGAC -3’; reverse: 5’-ATCGTCAGTACCGTTGACCTCAT-3’) were used to obtain DNA fragments by a PCR; these were individually inserted into the pGEM-T Easy vector (Promega, Madison, WI, USA). The inserted fragments were amplified with the T7 and SP6 primers by a PCR, and the respective products were used as templates for in vitro transcription with T7 or SP6 RNA polymerase (Roche) in the presence of digoxigenin (DIG)-UTP (Roche), to respectively synthesize the sense and antisense probes. The DIG-labeled RNA probes was run on a 1% agarose gel to check the size. The quality and concentrations of DIG-labeled RNA probes were determined using dot blot assays. For dot-blot assays, synthesized probes and standard RNA probes were spotted on nitrocellulose membrane according to the manufacturer’s instructions. After cross-linking and blocking, the membrane was incubated with an alkaline phosphatase-conjugated anti-dig antibody and stained with nitro blue tetrazolium (NBT) and 5-bromo-4-chloro-3-indolyl phosphate (BCIP).

Zebrafish embryos were anesthetized on ice and fixed with 4% paraformaldehyde in a phosphate-buffered saline (PBS; 1.4 mM NaCl, 0.2 mM KCl, 0.1 mM Na_2_HPO_4_, and 0.002 mM KH_2_PO_4_; pH 7.4) solution at 4°C overnight. Afterward, samples were washed with diethylpyrocarbonate (DEPC)-PBST (PBS with 0.1% Tween-20) several times (10 min/wash). Samples were subsequently incubated with hybridization buffer (HyB, 50% formamide, 5x SSC, and 0.1% Tween 20) at 65°C for 5 min, and then with HyB containing 500 μg/ml yeast transfer (t)RNA at 65°C for 4 h. Following overnight hybridization with 100 ng/ml of a DIG-labeled antisense or sense RNA probe, embryos were serially washed with 50% formamide-2x SSC (65°C for 20 min), 2x SSC (65°C for 10 min), 2x SSC (65°C for 10 min), 0.2x SSC (65°C for 30 min, twice), and PBST (room temperature for 10 min). Embryos were then immunoreacted with an alkaline phosphatase-coupled anti-DIG antibody (1:8000), and stained with NBT (Roche) and BCIP (Roche).

### Whole-mount immunocytochemistry

For triple in situ hybridization and immunocytochemistry, zebrafish samples were first hybridized in situ, and subsequently subjected to immunohistochemistry. After in situ hybridization, samples were washed with PBS, and incubated with 3% bovine serum albumin (BSA) for 2 h, before being incubated overnight at 4°C with an anti-avian NKA (α5) monoclonal antibody (diluted 1: 200) (Developmental Studies Hybridoma Bank) and an anti-killifish (*Fundulus heteroclitus*) HA polyclonal antibody (diluted 1: 100) [24]. Samples were subsequently incubated with Alexa Fluor 488 goat anti-mouse and Alexa Fluor 568 goat anti-rabbit antibodies for 2 h at room temperature. Images were obtained with an upright microscope (BX60; Olympus, Tokyo, Japan) equipped with a digital camera (Canon 50D, Tokyo, Japan).

### SIET and measurement of ionic gradients

The SIET was used to measure H^+^ at the skin and ionocyte surface of 3–4 dpf embryos. Glass capillary tubes (no. TW 150–4; World Precision Instruments, Sarasota, FL, USA) were pulled on a Sutter P-97 Flaming Brown pipette puller (Sutter Instruments, San Rafael, CA, USA) into micropipettes with tip diameters of 3~4 μm. These were then baked at 120°C overnight and coated with dimethyl chlorosilane (Sigma-Aldrich) for 30 min. The micropipettes were backfilled with a 1-cm column of electrolytes (40 mM KH_2_PO_4_ and 15 mM K_2_HPO_4_; pH 7) and frontloaded with a 20~30-μm column of liquid ion-exchange cocktail (H^+^ ionophore I cocktail B; Sigma-Aldrich) to create an ion-selective microelectrode (probe). Details of the system were described in previous reports [19, 25, 26, 27]. To calibrate the ion-selective probe, the Nernstian property of each microelectrode was measured by placing the microelectrode in a series of standard solutions (pH 6, 7, and 8 for the H^+^ probe). By plotting the voltage output of the probe against log [H^+^] values, a linear regression yielded a Nernstian slope of 58.6 ± 0.8 (*n* = 10) for H^+^. The SIET was performed at room temperature (26~28°C) in a small plastic recording chamber filled with 2 ml of recording medium. The recording medium contained NW, 300 μM MOPS buffer, and 0.1 mg/l ethyl 3-aminobenzoate methanesulfonate (tricaine; Sigma-Aldrich). The pH of the recording medium was adjusted to 7.0 by adding a NaOH or HCl solution.

Before the measurement, an anesthetized larva was positioned in the center of the chamber with its lateral side contacting the base of the chamber. To measure the H^+^ activity at the surface of the larva, the H^+^-selective probe was moved to the target position (~10 μm from the skin surface), voltages were recorded for 1 min, and the median value was used to calculate H^+^ activity. After recording at the skin surface, the probe was moved away from the skin (~10 mm) to record and calculate the background H^+^ activity. In this study, Δ[H^+^] was used to represent the H^+^ gradient between the target position and background. The gradient reflects the integrated H^+^ activity of skin cells (including keratinocytes and ionocytes) near the target position.

### Measurement of H^+^ gradients at specific cells

To record the surface H^+^ (Δ[H^+^]) concentration at specific cells, the probe was moved to a position 1~2 μm above the apical membranes of cells. The voltage difference in microvolts was measured by probing orthogonally to the surface at 10-μm intervals. Five replicates of recordings at an ionocyte or keratinocyte were performed, and the median value was used to calculate the Δ[H^+^] of the cell. Voltage differences obtained were converted into ionic gradients by ASET software following previous reports [19, 25]. To measure 1% CO_2_-induced acid secretion by the yolk-sac skin and individual ionocytes, embryos were pre-incubated in 1% CO_2_ NW for 10 min and then measured in NW without 1% CO_2_.

### Morpholino design and microinjection

Morpholino oligonucleotides (MOs) were obtained from Gene Tools (Philomath, OR, USA). The sequence of MOs against *aqp1a*.*1* was 5’-AAGCCTTGC TCT TCA GCT CGT TCA T-3’ which was shown to effectively suppress the expression of *aqp1a*.*1* in zebrafish [16]. A standard control MO (5’-ATCCATCTTGTGTGTTAGAAAACTG-3’) was also used as a control. The control MO provided by Gene Tools had no target and no significant biological activity. An MO solution was prepared with sterile water and contained 0.1% phenol red as a visualizing indicator. The MO was microinjected into embryos at the 1~4-cell stage with an IM-300 microinjector system (Narishige Scientific Instrument Laboratory, Tokyo, Japan). In preliminary tests, embryos injected with 4 ng of the control MO showed no significant differences in survival rates, morphology, or H^+^ gradients compared to WT embryos. Embryos injected with 4 ng of the aqp1a.1 MO had a normal morphology and survival rate.

### Drug preparation and treatment

P-Chloromercuribenzoate (PCMB; Sigma-Aldrich) was dissolved in distilled water to a stock concentration of 40 mM. Adequate stock was dissolved in NW or hypercapnic water to final concentrations of 200~500 μM. Thereafter embryos were measured in recording medium without drugs.

### Statistical analysis

Data are expressed as the mean ± standard error (SE; with *n*, number of embryos or ionocytes). Values from each condition were analyzed using a one-way analysis of variance (ANOVA) followed by Tukey’s pairwise comparisons. Student’s unpaired *t*-test (two-tailed) was used for simple comparisons of two means. Significance was set at a level of 0.05.

## Results

### Expression levels of *aqp* transcripts in gills and embryos

**Fig 1:**
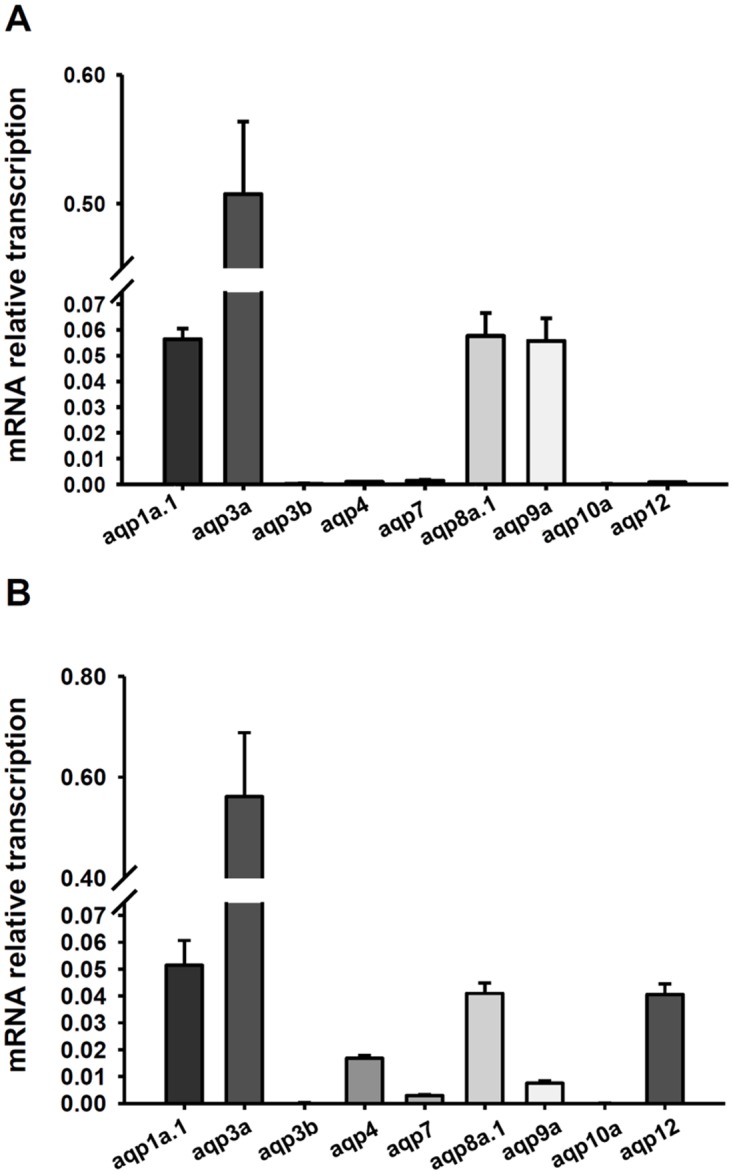
Gene expression of *aqps*. qPCR analysis of *aqp1a*.*1*, *aqp3a*, *aqp3b*, *aqp4*, *aqp7*, *aqp8a*.*1*, *aqp9a*, *aqp10a*, and *aqp12* mRNAs in gills (A) and 3-dpf zebrafish embryos (B). Values were normalized to *rpl13*. Values are expressed as the mean ± SE (*n* = 6).

**Fig 2:**
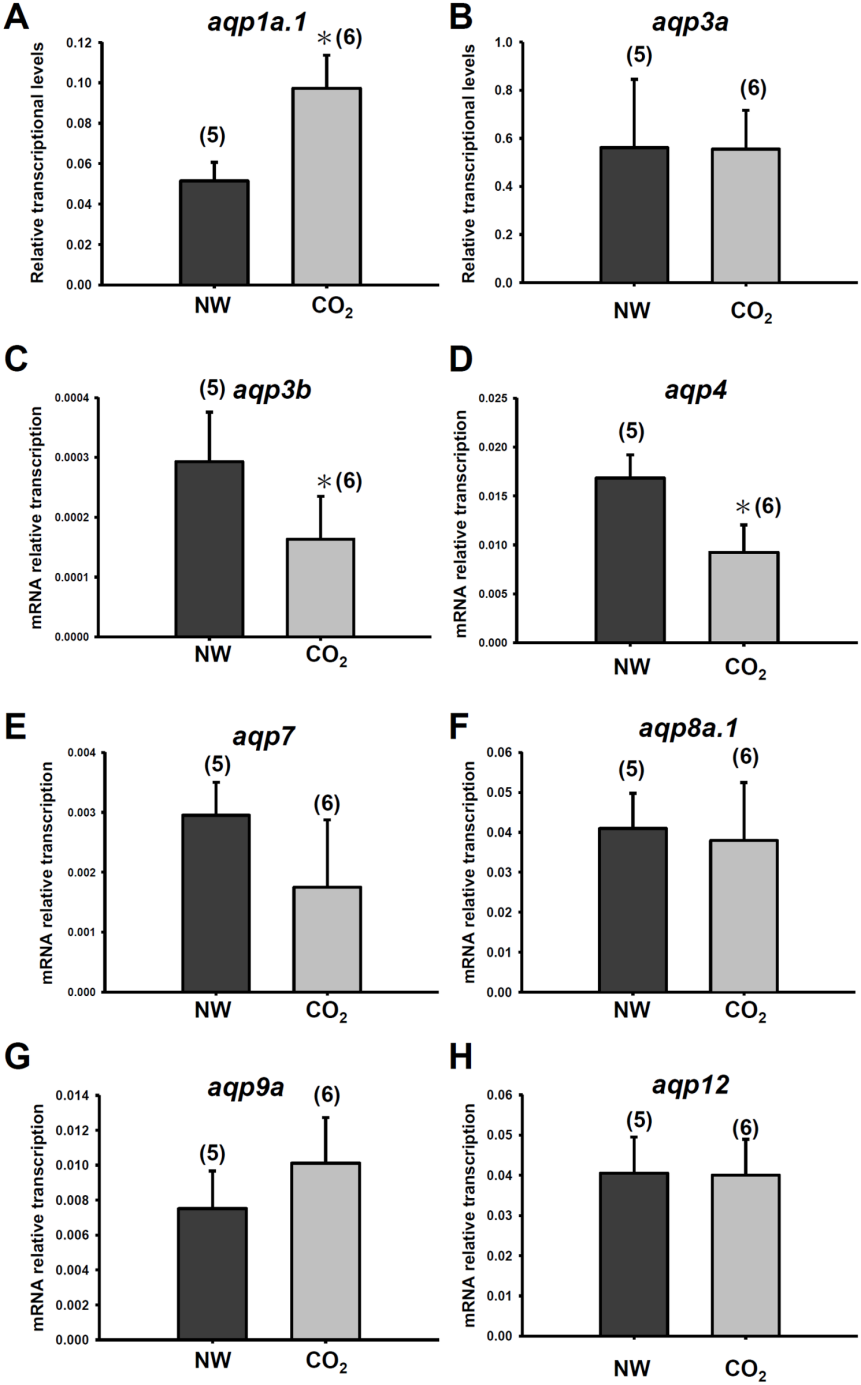
Gene expression of *aqps* in embryos exposed to hypercapnic water. qPCR analysis of *aqp1a*.*1* (A), *aqp3a* (B), *aqp3b* (C), *aqp4* (D), *aqp7* (E), *aqp8a*.*1* (F), *aqp9a* (G), and *aqp12* (H) mRNAs in embryos exposed to 1% CO_2_ for 3 days (0~72 dpf). Values were normalized to *rpl13*. Values are expressed as the mean ± SE. The number of samples is shown in parentheses. * Significant difference (Student’s *t*-test, *p*<0.05).

A previous study used an RT-PCR to show that *aqp1a*.*1*, *aqp3a*, *aqp3b*, *aqp4*, *aqp7*, *aqp8a*.*1*, *aqp9a*, *aqp10a*, and *aqp12* are expressed by zebrafish gills [28]. Herein, we further used a qPCR to determine mRNA levels of those *aqp*s in gills (Fig 1A). The results showed that *aqp1a*.*1*, *aqp3a*, *aqp8a*.*1*, and *aqp9a* in gills had relatively higher expression levels among those *aqp*s (Fig 1A); *aqp1a*.*1*, *aqp3a*, *aqp4*, *aqp8a*.*1*, and *aqp12* had higher expression levels in whole embryos (Fig 1B). After exposure to 1% CO_2_ (hypercapnia) for 3 days (0~72 h post-fertilization; hpf), expression of *aqp1a*.*1* was upregulated in embryos, whereas *aqp3b* and *aqp4* were downregulated (Fig 2). Because expression levels of *aqp3b* and *aqp4* were relatively low in gills and not known for CO_2_ permeation, we only investigated the function of *aqp1a*.*1* in the following experiments.

### Localization of *aqp1a*.*1* in skin ionocytes of larvae

**Fig 3:**
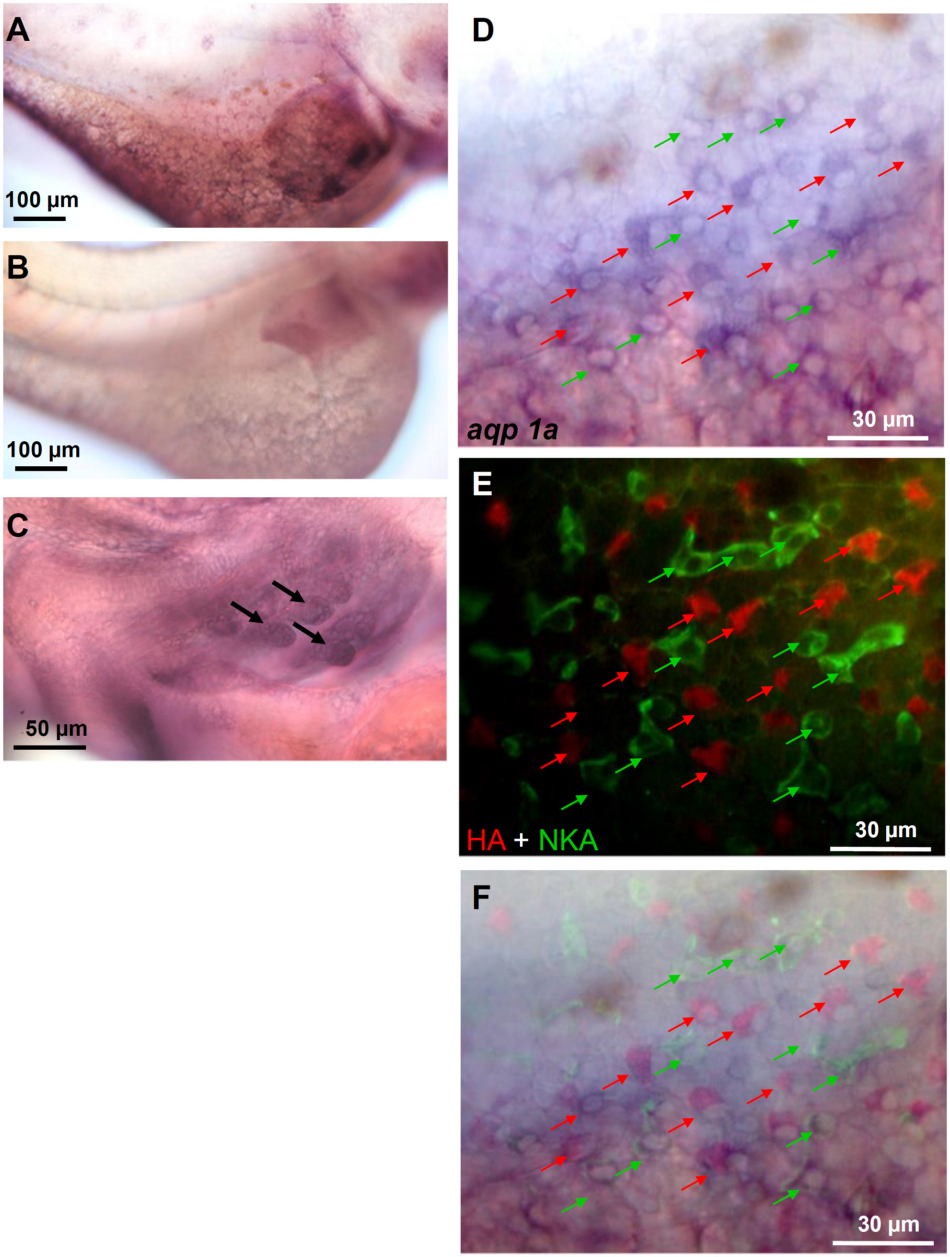
Localization of *aqp1a*.*1*. Triple-labeling of *aqp1a*.*1* mRNA (with in situ hybridization), H^+^-ATPase (HA), and Na^+^/K^+^-ATPase (NKA) (with immunofluorescence) in 3-dpf embryos. The *aqp1a*.*1* antisense probe labeled ionocytes in the yolk-sac skin of embryos (A, D). Those signals were not found in the negative control with an *aqp1a*.*1* sense probe (B). *aqp1a*.*1* signals (arrows) were also found in developing gills (C). The yolk-sac *aqp1a*.*1* signals (D) were co-localized with HA (red arrows in E) and NKA (green arrows in E). A merged image of (D) and (E) is shown in (F).

Localization of *aqp1a*.*1* was determined by in situ hybridization and triple-labeled with immunocytochemistry of NKA (a marker of NaR cells) and HA (a marker of HR cells). The *aqp1a*.*1* antisense probe labeled ionocytes that were dispersed through the yolk-sac skin (Fig 3A) and branchial region (Fig 3C) of 3-day post-fertilization (dpf) embryos. Those signals were not detected by a sense probe (negative control) (Fig 3B). Colocalization of *aqp1a*.*1* mRNA with HA or NKA proteins was found, which demonstrates that *aqp1a*.*1* is mainly expressed by both HR and NaR cells (Fig 3D–3F).

### Effect of *aqp1a*.*1* knockdown on CO_2_-induced acid-secretion from the yolk-sac skin of embryos

**Fig 4:**
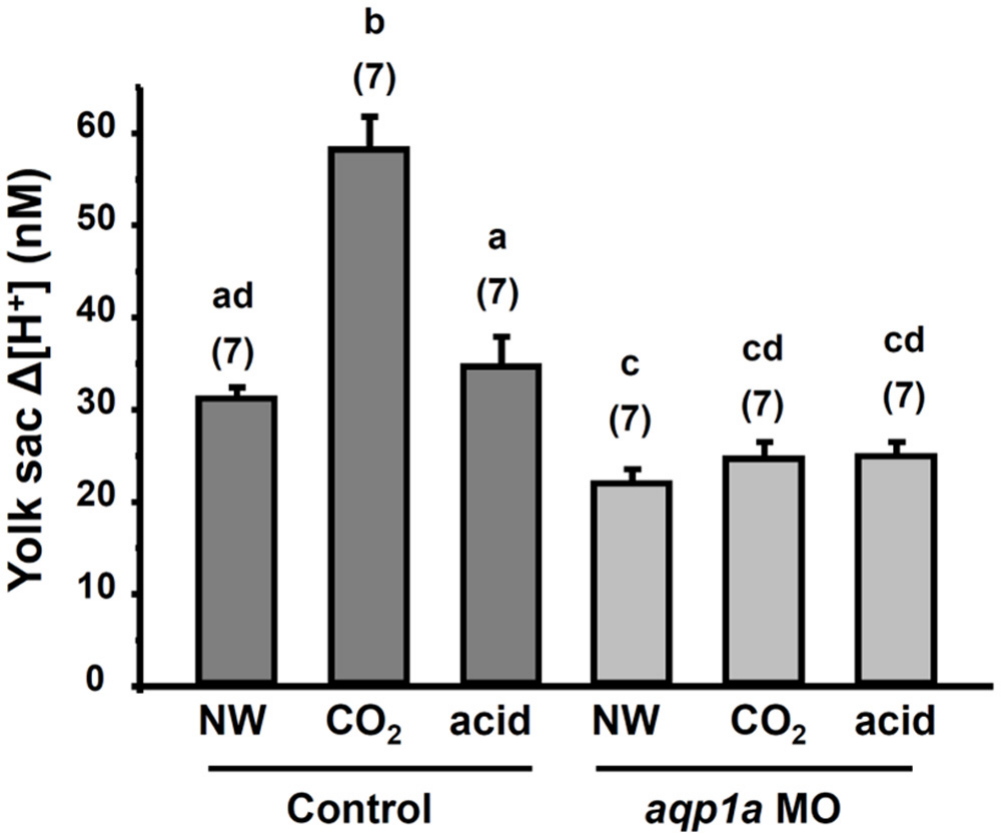
Effect of *aqp1a*.*1* knockdown on acid secretion by yolk-sac skin. The H^+^ gradient (Δ[H^+^]) at the yolk-sac skin of 3-dpf embryos injected with the control morpholino (Control MO) or *aqp1a*.*1* morpholino (*aqp1a*.*1* MO). Embryos were exposed to normal water (NW), 1% CO_2_ (CO_2_), and acidic water (acid) for 10 min before the SIET measurements. Data are presented as the mean ± SE. The number of analyzed embryos is shown in parentheses. ^a,b,c,d^ Significant difference (by one-way ANOVA and Tukey’s comparison; *p<*0.05).

The SIET was used to measure the H^+^ gradient (Δ[H^+^]) at the yolk-sac skin surface following our previous studies [17, 29]. The positive H^+^ gradient detected at yolk-sac skin indicates that acid is secreted from the yolk-sac skin (Fig 4). The H^+^ gradient in the CO_2_ group (embryos exposed to 1% CO_2_ for 10 min) was approximately 2-fold higher than that in the NW group, indicating that 1% CO_2_ incubation significantly increased acid secretion by skin cells (Fig 4). In contrast, embryos exposed to acid water (pH 6.3, normal CO_2_) for 10 min did not exhibit a significantly increased H^+^ gradient (Fig 4). The same measurements were also conducted in embryos injected with the *aqp1a*.*1* MO. The H^+^ gradient was significantly suppressed in those embryos; incubation in 1% CO_2_ or acid water did not significantly increase the H^+^ gradient in those embryos (Fig 4).

### Effect of *aqp1a*.*1* knockdown on CO_2_-induced acid-secretion from individual ionocytes

**Fig 5:**
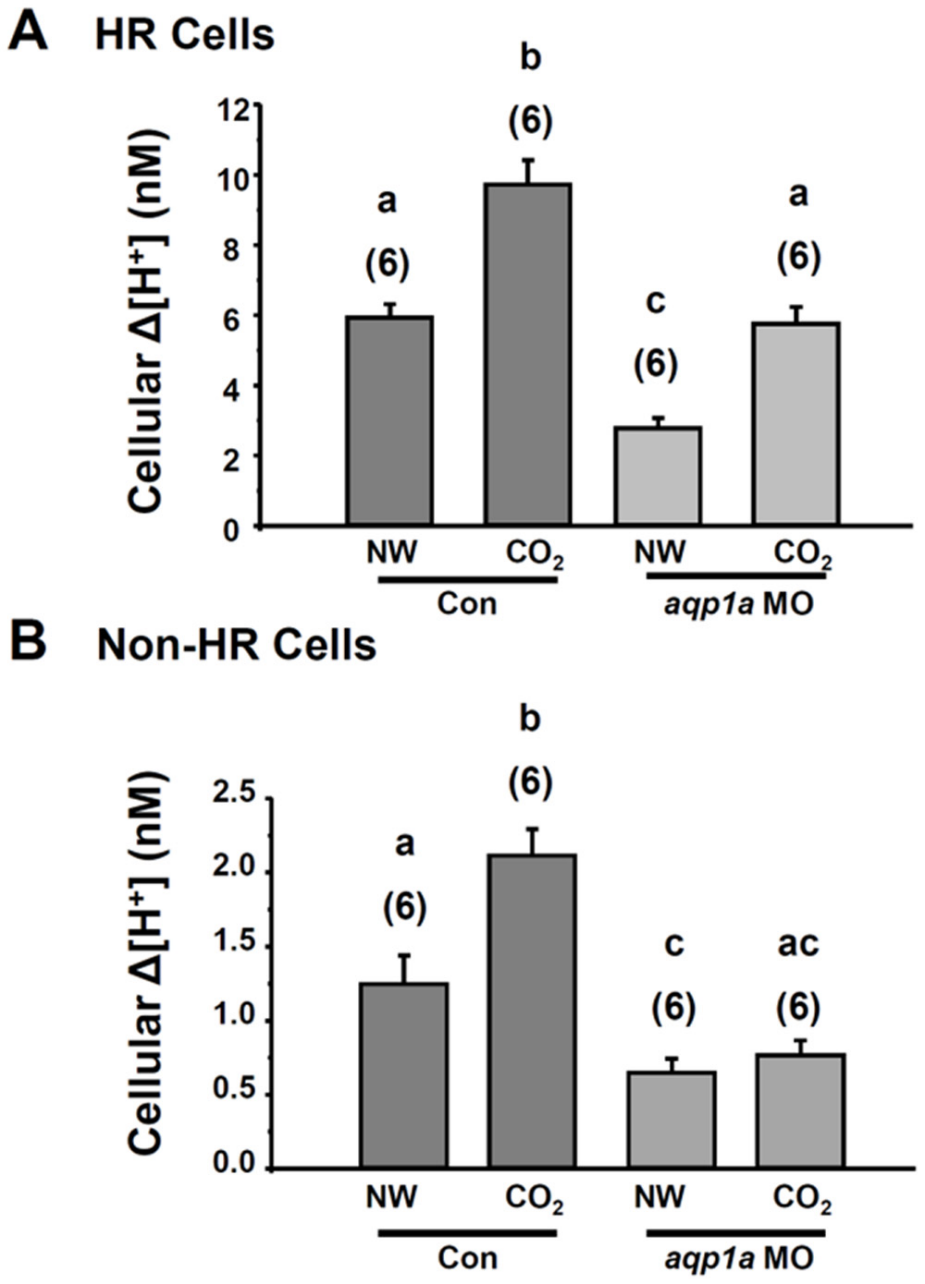
Effect of *aqp1a*.*1* knockdown on acid secretion by ionocytes. Effect of The H^+^ gradient (Δ[H^+^]) at the surface of HR cells (A) and non-HR cells (B) in 3-dpf embryos injected with the control morpholino (Control) or *aqp1a*.*1* morpholino (*aqp1a*.*1* MO). Embryos were exposed to normal water (NW) or 1% CO_2_ (CO_2_) for 10 min before the SIET measurements. Data are presented as the mean ± SE. The number of analyzed cells is shown in parentheses. ^a,b,c^ Significant difference (by one-way ANOVA and Tukey’s comparison; *p<*0.05).

Two groups of ionocytes, HR cells and non-HR cells, in the skin of embryos were distinguished by their morphology and distribution during SIET probing [18, 29]. As shown in our previous reports [18, 29], H^+^ gradients recorded at HR cells were remarkably higher than those recorded at non-HR cells (NW in Fig 5). After 1% CO_2_ incubation for 10 min, a significant increase in the H^+^ gradient was found at both HR cells and non-HR cells. Whereas morpholino knockdown significantly decreases H^+^ secretion by HR and non-HR cells of embryos (Fig 5A and 5B), CO_2_ treatment still increased H^+^ secretion in the HR cells but not in the non-HR cells (Fig 5B).

### Effect of a mercury compound on CO_2_-induced acid secretion from yolk-sac skin and HR cells

**Fig 6:**
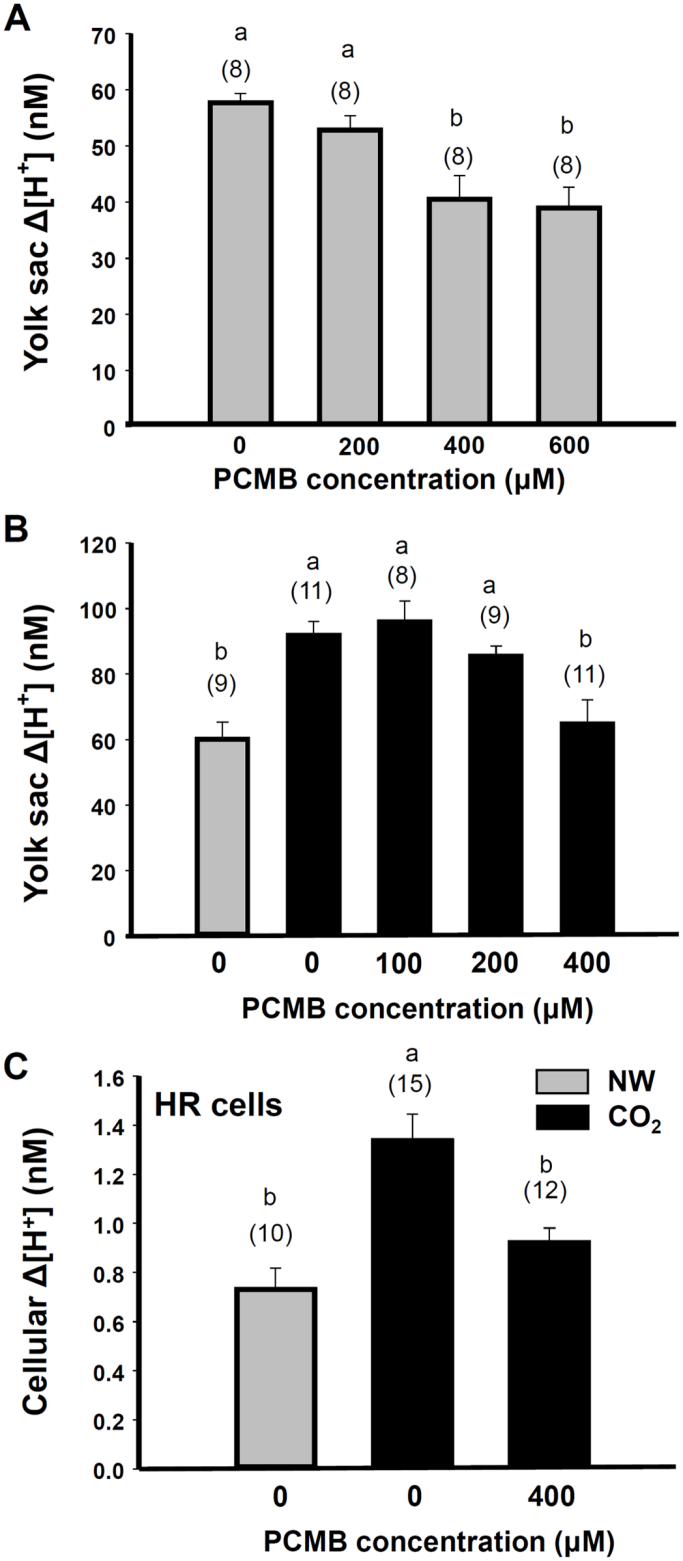
Effect of PCMB on acid secretion by the skin and ionocytes. Effect of PCMB on the H^+^ gradient (Δ[H^+^]) at the yolk-sac skin (A, B) and individual HR cells (C) in 4-dpf embryos with (black bars; CO_2_) or without (gray bar; normal) 1% CO2 pre-incubation for 10 min. Data are presented as the mean± SE. The number of samples (embryos in A; cells in B) is shown in parentheses. ^a,b,c,d^ Significant difference (by one-way ANOVA and Tukey’s comparison; *p<*0.05).

A mercury compound (PCMB) was used to block the function of AQP1 as shown in previous studies [15, 30]. H^+^ gradients at the yolk-sac skin and individual HR cells were measured in embryos with or without pre-incubation in 1% CO_2_ water. PCMB produced a dose-dependent inhibition of the skin H^+^ gradients in normal embryos (without CO_2_ pre-incubation, Fig 6A). H^+^ gradients at the yolk-sac skin increased significantly after 1% CO_2_ incubation (black bar; 0 PCMB; Fig 6B) and the increase was significantly inhibited by 400 μM PCMB treatment (gray bar: without CO_2_; black bar: with CO_2_; Fig 6B). A similar result was found in the H^+^ gradient measured at HR cells (Fig 6C).

## Discussion

In the zebrafish, mRNAs of AQP1a (1a.1), -3a, -3b, -4, -7, -8aa (8a.1), -9a, -10a, and -12 were identified in gills with an RT-PCR [28]. Herein, we further used a qPCR to precisely compare mRNA levels of those isoforms in zebrafish gills and found that AQP1a.1, -3a, -8a.1, and -9a were dominant isoforms (Fig 1A). In previous studies, AQP 3 was identified in gills of several euryhaline fish species including sea bass (*Dicentrarchus labrax*) [31], salmon (*Salmo salar*) [32], medaka (*Oryzias dancena*) [33], killifish (*Fundulus heteroclitus*) [34], and eel (*Anguilla Anguilla*) [35]; AQP1 was identified in gills of climbing perch (*Anabas testudineus*) [36], sea bass (*D*. *labrax*) [31], salmon (*S*. *salar*) [32], medaka (*O*. *dancena*) [33], and zebrafish (*Danio rerio*) [28]. Expression levels of AQP1 and -3 in gills were examined in several fishes subjected to salinity changes. mRNA and/or protein levels of AQP3 in most examined species declined after transfer from fresh water to seawater [31, 32, 33, 34, 35]. The mRNA level of AQP1 in salmon and medaka declined after transfer from fresh water to seawater [32, 33]. However, no significant difference was found in mRNA levels of AQP1 between seawater- and fresh water-acclimated climbing perch and sea bass [31, 36]. Given this, there seems no convincing and consistent evidence to clarify the roles of AQP1 and -3 in fish gills.

Both AQP1 and AQP3 are broadly expressed in various fish tissues including gill, kidney, intestine and skin [28, 33, 37]. In this study, the mRNA expression level of AQP3a was much higher than that of AQP1a.1 in gills and whole embryos (Fig 1). A similar result of AQP3 and AQP1 was reported in gills of sea bass acclimated to fresh water [31]. The abundant expression of AQP3 indicates that it may play a critical role in transporting H_2_O in fish tissues. Unlike AQP1 which is permeable to both H_2_O and CO_2_, AQP3 is permeable to H_2_O but not CO_2_ [2, 3, 4]. Our result shows that AQP3a and AQP3b were not induced by CO_2_ incubation (Fig 2), suggesting that AQP3 is not involved in CO_2_ transport.

Cellular localization of AQP3 in fish gill was investigated in several fish species. In salmon (*Oncorhynchus nerka*), the AQP3 protein was localized to ionocytes of gills [38]. However, the AQP3 mRNA/protein was localized to both pavement cells and ionocytes in the European (*A*. *anguilla*) and Japanese eel (*A*. *japonica*) [35, 39]. AQP1a.1 mRNA was first identified in skin ionocytes of zebrafish embryos by Chen [15], and its protein was localized to basolateral membranes of ionocytes in zebrafish embryos [16]. In the present study, triple *in situ* hybridization and immunocytochemistry was conducted to show mRNA localization of AQP1a.1 in two subtypes of ionocytes, HR cells and NaR cells, supporting protein localization in the previous study.

Kwong and colleagues [16] showed that knockdown of AQP1a.1 expression in zebrafish embryos reduced water movement across the embryonic skin, suggesting that AQP1a.1 is able to facilitate transcellular water transport. However, they did not show how AQP1a.1-knockdown influences the function of ionocytes. Herein, we found that the mRNA level of AQP1a.1 in zebrafish embryos was induced after exposure to 1% CO_2_ for 3 days. Most importantly, knockdown of AQP1a.1 with a morpholino injection or treatment with an AQP inhibitor remarkably suppressed acid secretion by skin HR cells after 1% CO_2_ incubation for 10 min (Figs 4, 5 and 6), suggesting that AQP1a.1 plays a critical role in facilitating acid secretion by HR cells.

In mammals, the kidneys control and maintain the systemic acid-base status by three intricately linked mechanisms: the reabsorption of filtered bicarbonate, the excretion of acids or alkali, and the de novo generation of bicarbonate [40]. For bicarbonate generation and acid secretion, interstitial CO_2_ passes into proximal tubule cells and intercalated cells from the basolateral membrane and reacts with intracellular water to form HCO_3_^−^ and H^+^. Then, the H^+^ is excreted apically by H^+^-ATPase or the NHE, and HCO_3_^−^ enters the interstitium via the basolateral anion exchanger [40, 41]. Animal studies in AQP1^−/−^ mice showed that 50% less HCO_3_^−^ reabsorption was observed in isolated proximal tubules. AQP1^−/−^ mice also had a decreased ability to resist acid loads [11]. Those studies suggested that AQP1 in the kidneys plays an important role in acid-base regulation.

**Fig 7:**
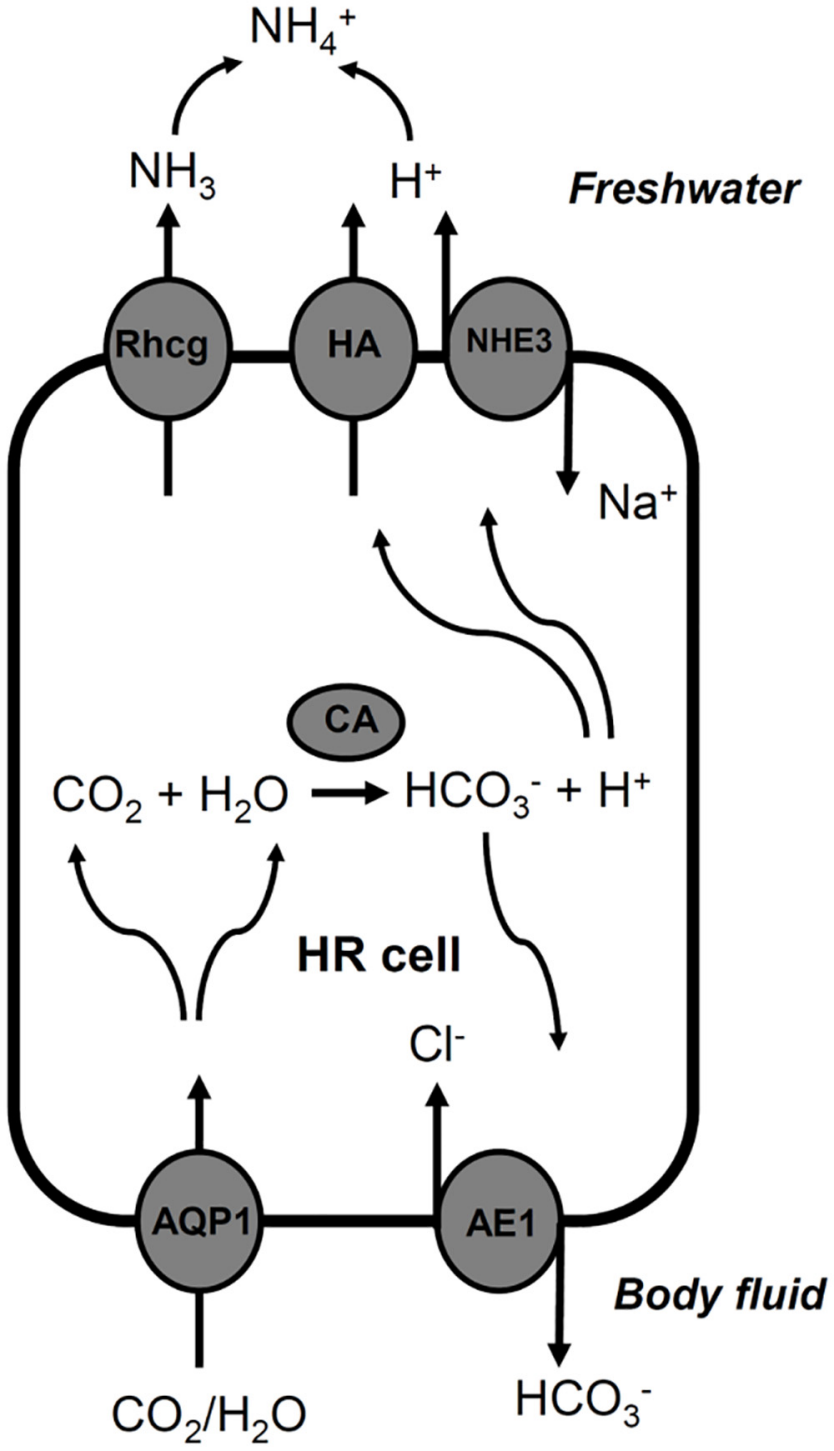
The proposed role of AQP1a.1 in acid-secreting HR cells of zebrafish [modified from 13, 14]. Refer to the text for detail. AQP1, aquaporin 1a.1; AE1, anion exchanger 1b; CA2, carbonic anhydrase 2; HA, H^+^-ATPase; NHE3, Na^+^/H^+^ exchanger 3; Rhcg1, Rhesus C glycoprotein 1.

An acid secretion mechanism similar to that found in mammalian kidney was found in fish gill/skin ionocytes [42, 43]. In the zebrafish, HR cells are the major subtype of ionocytes for H^+^ secretion and HCO_3_^−^ reclamation, and H_2_O and CO_2_ are generally believed to be the major source for generating H^+^ and HCO_3_^−^ [14, 17, 29]. The expression of AQP1a.1 by HR cells was thus hypothesized to provide higher permeability of cell membranes to CO_2_ and thus increase the production of H^+^ and HCO_3_^−^ in HR cells. This hypothesis is supported by the present results that knockdown of AQP1a.1 and PCMB treatment suppressed acid secretion of embryos (Figs 4 and 5). The model of acid secretion by HR cells is illustrated in Fig 7 [modified from 13, 14]. AQP1a.1 in the basolateral membrane can permeabilize and increase CO_2_ supply intracellularly, therefore carbonic anhydrase (CA2) can generate H^+^ and HCO_3_^−^ to supply H^+^ for the apical H^+^-ATPase and Na^+^/H^+^ exchanger (NHE3) to produce acid secretion. The HCO_3_^−^ would thus be transported to the inner environment through the basolateral AE1b of HR cell.

In this study, knockdown of AQP1a.1 suppressed roughly half of acid secretion by the embryos (Figs 4 and 5). This is consistent with findings in mammalian knockouts of AQP1, where renal acid transport is inhibited by approximately 50%, compared to wild type animals [11]. After morpholino knockdown, 10 min CO_2_ incubation still increased H^+^ secretion in HR cells (Fig 5A), suggesting that transmembrane CO_2_ permeation is also a possible pathway thus H^+^ secretion can be elevated by CO_2_ provided through this pathway after knockdown of AQP1a.1.

Although, H^+^ secretion by non-HR cells was much less than that by HR cells, it was slightly elevated after 10 min of 1% CO_2_ exposure and the increase was totally suppressed by AQP1a.1 knockdown (Fig 5). Since AQP1a.1 is also expressed by Ca^2+^-absorbing NaR cells (the major portion of recorded non-HR cells), elevation of H^+^ secretion by NaR cells after CO_2_ loading was supposedly due to internal CO_2_ passing though NaR cells [15], and apparently AQP1a.1 in NaR cells can enhance this process. Unlike HR cells, NaR cells are not powerful acid-secreting cells, and thus AQP1a.1 in NaR cells should not be mainly for acid secretion but for other functions such as maintaining cell volume balance [16]. Further investigation of the role of AQP1a.1 in NaR cells is required to fully understand the physiology of AQP in ionocytes.
